# Predictive factors of poor outcome in road traffic injures; a retrospective cohort study

**Published:** 2017-01-09

**Authors:** Hamid Reza Hatamabadi, Majid Shojaee, Parvin Kashani, Mohammad Mehdi Forouzanfar, Dorrin Aghajani Nargesi, Mohammad Reza Amini Esfahani

**Affiliations:** 1 Safety Promotion and Injury Prevention Research Center, Shahid Beheshti University of Medical Sciences, Tehran, Iran.; 2 Department of Emergency Medicine, Shahid Beheshti University of Medical Sciences, Tehran, Iran.; 3 Department of Emergency Medicine, Shiraz University of Medical Sciences, Shiraz, Iran.

**Keywords:** Accidents, traffic, hospitalization, patient outcome assessment, epidemiology

## Abstract

**Introduction:**

Road traffic injuries (RTI) are among the most important health problems worldwide as they cause more than 1.2 million deaths and 50 million injuries each year. Therefore, the present study aims to evaluate the outcome and aftermath of RTI in those who were injured and hospitalized due to a traffic accident.

**Methods:**

In the present retrospective cohort study with a one-year follow-up, data were extracted from the profiles of the RTI hospitalized patients. Outcome of the patients was evaluated at the time of discharge and 1-year later including their living state, presence of a disability or complete recovery.

**Results:**

1471 patients were studied (mean age of 32.8±17.0; 80.3% male). 571 (38.8%) had mild disability, 684 (46.5%) moderate disability, and 85 (5.8%) had severe disability at the time of discharge. In the end, 53 (3.6%) died. In the 1-year follow-up, 194 (13.2%) had mild disability, 43 (2.9%) had moderate disability, 9 (0.6%) had severe disability, and 7 (0.5%) were in a vegetative state. Presence of an underlying disease (p=0.03), loss of consciousness for more than 24 hours (p=0.04), spinal injury (p=0.002), presence of multiple trauma (p=0.01), increased ISS (p<0.001), need for ventilator (p<0.001), and organ injuries during hospitalization (p<0.001) are independent factors that increase the risk of poor outcome in RTI patients.

**Conclusion:**

Based on the results of the present study, underlying illnesses, loss of consciousness for more than 24 hours, spinal injury, multiple trauma, increased ISS, need for ventilator, and organ injuries during hospitalization were independent factors that increased the probability of poor outcome in RTI injuries.

## Introduction

Road traffic injuries (RTI) are among the most important health problems worldwide as they cause more than 1.2 million deaths and 50 million injuries each year. More than 90% of mortalities due to RTI occur in low and middle income countries (1, 2). It is predicted that in the next 5 years RTI will lead to 6 million deaths and 60 million injuries, only in developing countries. In 1990, RTI ranked 9^th^ in the most important factors determining population health and it is predicted to become the 3^rd^ cause of mortality and disability by 2020. The reports also show that 50% of the dead were 15-43 years old, who are the most effective people in a society's financial development (3). In Iran RTI rate is very high and fatal RTI rate is 33 in 100000 people, which emphasizes the need for more research and taking preventive measures and efficient treatment in managing RTI (-).

The high social and financial costs of RTI and its physical and mental side effects on people and societies is the major problem that transportation managers and health providers must face. This challenge is many times more in developing countries, where RTI rate is increasing and its direct and indirect costs are more than the developed countries. World Bank report shows that the number of people who die of RTI in Iran has increased by 10%, which is higher than most developing countries and is very undesirable and worrisome compared to world standards (2).

In its last report, World Health Organization has expressed the need for more research on the epidemiologic pattern of RTI in low and middle income countries to determine the dimensions of the problem and identify those who are most susceptible to RTI, since no accurate estimation exists regarding the social and economic effects of RTI in these countries. Although valuable efforts have been made to identify the effects and outcomes of RTI in Iran in recent years, there is still a shortage of available data in this regard (-). Therefore, the present study aims to retrospectively evaluate the outcome and aftermath of RTI in those who were injured and hospitalized due to a traffic accident. 

## Methods

***Study design***

The present study is a retrospective cohort one, with a one-year follow-up, carried out in two educational hospitals in Tehran, Iran. Patients referred to the hospitals from April 2012 to March 2013 were included and Ethics Committee of Shahid Beheshti University of Medical Sciences approved the study. Data collection forms were anonymous and patient data remained confidential.

***Participants***

The studied population consisted of all the patients injured in RTI over the course of the study, who had an accident with at least 1 vehicle. Patients with incomplete or unreachable data were excluded. There was no age and sex limitation.

***Data collection***

Data collection was done using a checklist that consisted of demographic data (age, sex, level of education), trauma mechanism, type and location of injury, type of vehicle in accident, route of transport to emergency department (ED) (by ambulance, taxi, or personal vehicle), clinical measures taken in pre-hospital, hospitalization status, hospitalization duration, intensive care unit admission, injury severity score (ISS), need for ventilator, organ failure, and outcomes (death, disability, or complete recovery at the time and one year after discharge). Data were gathered by trained emergency medicine residents. Their trainings consisted of research tools management (how to fill a checklist, data recording) and summarizing medical data.

Data were extracted from the patients’ profiles and quality of data collection was evaluated by the head researcher of each hospital every 24 hours. In addition, at the end of each week, some checklists were randomly chosen and their quality was controlled by the chief researcher to ensure the quality of data collection. In this study, injury severity was classified into 4 groups: mild (ISS < 9), moderate (ISS 9-15), severe (ISS 16-25), and profound (ISS > 25).

***Outcomes***

Living status (dead or alive), and disability or complete recovery at the time of discharge were appraised, and in-hospital complications such as embolism, deep vein thrombosis, infection, organ failure, need for ventilator, infection, high or low blood pressure, hypothermia, hypoxia, seizure, sepsis and shock were evaluated. Death and severe disability were considered as poor outcome. Glasgow outcome scale (GOS) was used for the 1-year follow-up outcome evaluation (panel 1). GOS divides patients into 2 groups based on desirable and undesirable outcome: poor consisting of GOS score 1-3 and desirable with the score of 4-6. In the 1-year follow-up, the patient or their relatives were contacted by phone. Cases that could not be contacted after calling 3 times (due to not responding, wrong number or the phone number being sold) were considered as loss to follow-up.

**Panel 1:** Glasgow outcome scale

**Database**	**Search terms**
**1. Death**	Severe injury or death without recovery of consciousness
**2.** **Persistent vegetative state**	Severe damage with prolonged state of unresponsiveness and a lack of higher mental functions
**3. Severe disability**	Severe injury with permanent need for help with daily living
**4. Moderate disability**	No need for assistance in everyday life, employment is possible but may require special equipment.
**5. mild disability**	Light damage with minor neurological and psychological deficits.
**6. Good recovery**	Resumption of normal activities even though there may be minor neurological or psychological deficits.

**Table 1:** Relationship between demographic data and baseline characteristics of patients and their 1-year outcome

Factor	Desirable outcome	Poor outcome	Total	P
Age (mean ± SD)	32.0 ± 16.5	40.0 ± 21.5	32.8 ± 17.0	< 0.001
Sex (n, %)				
**Male **	1126 (80.3)	55 (79.7)	1181 (80.3)	0.90
**Female **	276 (19.7)	14 (20.3)	290 (19.7)	
Reference by (n, %)				
**Ambulance**	1003 (71.5)	45 (65.2)	1048 (71.2)	0.001
**Personal vehicle**	199 (14.2)	4 (5.8)	203 (13.8)	
**Referral from another hospital**	200 (14.3)	20 (29.0)	220 (15.0)	
Time before arrival (mean ± SD)	32.9 ± 20.3	33.5 ± 16.0	33.1	0.85
Trauma mechanism (n, %)				
**Automobile **	1024 (73.0)	39 (56.5)	1063 (72.3)	0.03
**Motorcycle **	153 (10.9)	11 (16.0)	154 (10.5)	
**Collision with a stationary object**	187 (13.3)	16 (23.2)	203 (13.8)	
**Bicycle **	12 (0.1)	0 (0.0)	12 (0.7)	
**Unknown**	36 (2.6)	3 (4.3)	39 (2.7)	
History of illness (n, %)				
**No**	1263 (90.1)	0 (0.0)	1263 (85.9)	
**Diabetes **	41 (3.0)	4 (5.8)	45 (3.1)	0.11
**Hypertension **	59 (4.1)	58 (84.1)	117 (7.9)	0.11
**Ischemic heart disease or stroke**	37 (2.7)	4 (5.8)	41 (2.8)	0.08
**Myocardial infarction**	2 (0.1)	2 (2.9)	4 (0.3)	0.009
**Cerebral Vascular Accident**	0 (0.0)	1 (1.4)	1 (0.1)	0.04
History of drug use (n, %)				
**No **	1278 (90.9)	49 (71.0)	1327 (90.2)	< 0.001
**Yes**	124 (9.1)	20 (29.0)	144 (9.8)	
Drug abuse (n, %)				
**No**	1134 (80.9)	53 (76.8)	1187 (80.7)	0.43
**Cigarette **	129 (9.2)	9 (14.8)	138 (9.4)	0.16
**Alcohol **	23 (1.6)	0 (0.0)	23 (1.6)	0.62
**Drugs **	103 (7.4)	6 (9.8)	109 (7.4)	0.49
**Hookah **	3 (0.2)	0 (0.0)	3 (0.2)	0.72
**Psychotropic drugs**	10 (0.7)	0 (0.0)	10 (0.7)	0.99

**Table 2:** Relationship between clinical factors of patients and their 1-year outcome

Factor	Desirable outcome	Poor outcome	Total	P
Life-threatening signs on admission (n, %)				
**No**	1286 (91.7)	65 (94.2)	1351 (91.8)	0.43
**Airway obstruction**	10 (0.7)	0 (0.0)	10 (0.7)	0.99
**Respiratory problems**	35 (2.5)	0 (0.0)	35 (2.4)	0.41
**Circulation problems**	71 (5.1)	4 (6.8)	75 (5.1)	0.99
Pupil (n, %)				
**Normal**	1120 (98.9)	51 (96.2)	1171 (98.7)	0.22
**Single-sided pupil dilation**	8 (0.7)	1 (1.9)	9 (0.8)	
**Double-sided mydriasis pupil dilation**	5 (0.4)	1 (1.9)	6 (0.5)	
Glasgow coma scale (n, %)				
**14-15**	1309 (93.4)	63 (91.3)	1372 (93.3)	0.34
**9-13**	55 (3.9)	2 (2.9)	57 (3.9)	
**> 9**	38 (2.7)	4 (5.8)	42 (2.8)	
Head trauma (n, %)				
**No**	1085 (77.4)	39 (54.5)	1124 (76.4)	< 0.001
**Yes**	317 (22.6)	30 (43.5)	347 (23.6)	
Loss of consciousness duration (n, %)				
**No**	1274 (95.5)	47 (81.0)	1321 (94.9)	< 0.001
**< 6 hours**	53 (4.0)	1 (1.7)	54 (3.9)	
**6-24 hours**	1 (0.1)	0 (0.0)	1 (0.1)	
**> 24 hours**	6 (0.4)	10 (17.3)	16 (1.1)	
Amnesia (n, %)				
**No **	1218 (92.4)	47 (83.0)	1265 (91.9)	< 0.001
**< 6 hours**	92 (7.0)	3 (5.2)	95 (6.9)	
**6-24 hours**	2 (0.2)	0 (0.0)	2 (0.2)	
**>24 hours**	6 (0.4)	8 (13.8)	14 (1.0)	
Site of injury (n, %)				
**Neck **	56 (4.1)	5 (7.2)	61 (4.4)	0.1
**Face **	16 (1.2)	0 (0.0)	16 (1.1)	0.99
**Chest **	111 (8.4)	12 (17.4)	123 (8.8)	< 0.001
**Abdomen and hip**	112 (8.5)	12 (17.4)	124 (8.9)	< 0.001
**Spine **	61 (4.6)	10 (14.5)	71 (5.1)	< 0.001
**Upper extremities**	247 (18.6)	8 (11.6)	255 (18.3)	0.20
**Lower extremities**	724 (54.6)	22 (31.9)	746 (53.4)	0.001
Multiple trauma (n, %)				
**No**	1180 (84.2)	40 (58.0)	1220 (82.9)	< 0.001
**Yes**	222 (15.8)	29 (42.0)	251 (17.1)	
Injury severity score (n, %)				
**< 9 (Mild)**	628 (52.4)	3 (4.5)	631 (49.9)	< 0.001
**9-15 (Moderate)**	338 (28.2)	5 (7.6)	343 (27.1)	
**16-25 (Severe)**	160 (13.4)	24 (36.4)	184 (14.6)	
**> 25 (profound)**	72 (6.0)	34 (51.5)	106 (8.4)	

**Table 3:** Relationship of therapeutic measures and side effects during hospitalization with patients' 1-year outcome

Variable*	Desirable outcome		Poor outcome	Total	p
Pre- hospital emergency measures (n, %)					
**Serum therapy**	1037 (74.5)		50 (72.5)	1087 (73.9)	0.71
**Intubation **	1 (0.06)		0 (0.0)	1 (0.1)	0.99
**Oxygen therapy**	765 (55.0)		46 (66.7)	811 (55.1)	0.06
**Neck collar**	443 (31.9)		33 (47.8)	476 (32.4)	0.006
**Back board**	326 (23.5)		31 (44.9)	357 (28.2)	< 0.001
**Splinting**	571 (41.1)		26 (37.7)	597 (40.6)	0.57
In- hospital emergency measures (n, %)					
**Blood transfusion**	149 (10.6)		40 (57.0)	189 (12.8)	< 0.001
**Cardiopulmonary resuscitation**	2 (0.14)		51 (73.9)	53 (3.6)	< 0.001
**Chest tube**	50 (3.6)		19 (27.5)	69 (4.7)	< 0.001
**Diagnostic peritoneal lavage**	19 (1.4)		23 (33.3)	42 (2.8)	< 0.001
**Need for ventilator**	48 (3.4)		50 (77.5)	98 (6.7)	< 0.001
Organ injury (n, %)					
**No**		1355 (96.5)	35 (50.1)	1390 (94.5)	< 0.001
**Respiratory **		1 (0.07)	5 (7.4)	6 (0.4)	< 0.001
**Coagulation abnormality**		1 (0.07	5 (7.4)	6 (0.4)	< 0.001
**Liver **		0 (0.0)	1 (1.5)	1 (0.1)	0.05
**Cardiovascular **		2 (0.1)	6 (8.8)	8 (0.6)	< 0.001
**Kidney**		4 (0.3)	4 (5.9)	8 (0.6)	< 0.001
**Sepsis **		1 (0.07)	4 (5.9)	5 (0.3)	< 0.001
**Infection **		26 (1.9)	8 (11.6)	34 (2.3)	< 0.001
**Embolism **		10 (0.7)	1 (1.5)	11 (7.5)	0.41
**Deep vein thrombosis**		2 (0.1)	0 (0.0)	2 (0.2)	0.99

*, Some patients have more than one injury or underwent more than one procedure.

**Table 4:** Independent effective factors on 1-year outcome of RTI patients

Variable	Regression coefficient	95% confidence interval	P
**Underlying illness **	1.15	0.11 – 2.34	0.03
**Loss of consciousness > 24 hours**	0.62	0.02 – 1.22	0.04
**Abdominal trauma**	-2.62	-4.49 - -0.74	0.006
**Spinal trauma**	1.98	0.75 – 3.21	0.002
**Multiple trauma **	1.02	0.10 – 2.14	0.01
**Increased** **injury severity score**	0.17	0.10 – 0.25	< 0.001
**Intensive care unit hospitalization**	1.98	-3.81 – 0.19	0.03
**Need for ventilator**	3.22	2.0 – 4.45	< 0.001
**Organ injuries during hospitalization**	3.69	2.16 – 5.21	< 0.001

**Figure 1:**
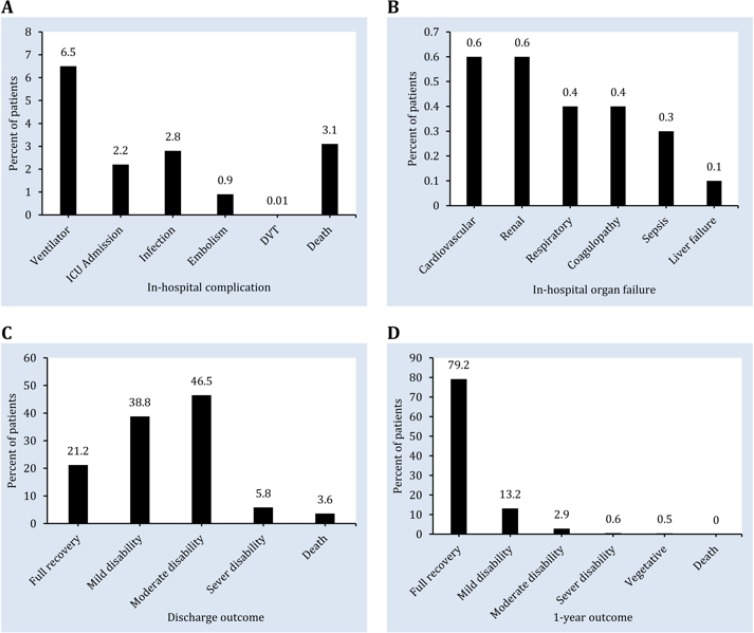
Patient outcomes. A) In-hospital complication; B) Organ injuries during hospitalization; C) Outcome of the patients at the time of discharge; D) 1-year outcome of the patients

***Statistical analyses***

Data were analyzed using STATA 11.0. Quantitative data were reported as mean and standard deviation and qualitative ones as frequency and percentage. Outcome (death, disability, complete recovery) and complications were assessed based on demographic data, baseline characteristics and clinical information. The association of each variable with 1-year outcome was then determined using independent t-test, chi square and exact Fisher's test. Finally, to assess the independent predictive factors of patients’ outcome, stepwise multivariate logistic regression analysis was used. In all analyses, p < 0.05 was considered as significance level.

## Results

***Baseline characteristics of the patients***

1941 patients were included in this study, 206 (10.6%) of which were discharged against medical advice and there were 264 (15.2%) cases of loss to 1-year follow-up. Therefore no data was available regarding their outcome. Analyses were done on the remaining 1471 patients. Their mean age was 32.8 ± 17.0 years ranging from 1 to 91 years (80.3% male). Tables 1 and 2 show the patients' demographic data, baseline characteristics and patients’ clinical variables. The 18-29 years age group had the most frequency with 657 (37.9%) patients. Most of the patients (71.2%) were referred to the hospital by an ambulance. 

Trauma mechanism was car accident in 1063 (72.3%) patients. Urban areas were the most common location with 43.5%. Glasgow coma scale (GCS) was between 14 and 15 in 1372 (93.3%) of the patients, 9-13 in 57 (3.9%), and < 9 in 42 (2.8%) cases. This loss of consciousness lasted less than 6 hours in 54 (3.9%), 6-24 hours in 1 (0.1%) and more than 24 hours in 16 (1.1%) patients. Lower extremities injury (53.4%) was the most common injury. 

***Patient outcomes***

Mean length of stay was 8.7 ± 8.3 days ranging from 1 to 96 days. 38 (2.6%) patients were hospitalized in the intensive care unit (ICU). Mean hospitalization duration in ICU was 7.7 ± 9.1 days (ranged 1-52 days). 17 (1.2%) of the patients were affected with wound infection, 6 (0.4%) with pulmonary embolism 8 (0.6%) with fat embolism, and 2 (0.2%) had deep vein thrombosis. In evaluating in-hospital organ failure, 6 (0.4%) cases of respiratory diseases, 6 (0.4%) cases of coagulation abnormalities, 1 (0.1) patient with liver problem, 8 (0.6%) patients with cardiovascular diseases, 8 (0.6%) with kidney diseases, and 5 (0.3%) with sepsis were observed (Figure 1A-B and Table 3).

Out of the 1471 studied patients, 312 (21.2%) were discharged with full recovery, while 571 (38.8%) had mild disability, 684 (46.5%) had moderate disability, and 85 (5.8%) had severe disability at the time of discharge. In the end, 53 (3.6%) patients died (Figure 1C).

After 1 year, 1165 (79.2%) patients had fully recovered, 194 (13.2%) had mild disability, 43 (2.9%) had moderate disability, 9 (0.6%) had severe disability, and 7 (0.5%) were in a vegetative state. No cases of death were reported during this time (Figure 1D). 

**Predictive factors of 1-year outcome:**

***Univariate analyses***

Higher ages (p < 0.001); being referred from another hospital (p = 0.001); high energy trauma mechanism (p = 0.03); having a history of myocardial infarction (p = 0.009), cerebral vascular accident (p = 0.04), drug use (p < 0.001); using neck collar (p = 0.006) and back board (p < 0.001) at pre-hospital setting; having head trauma (p< 0.001); the longer duration of loss of consciousness (p <0.001); need for ventilator (p < 0.001); hospitalization in ICU (p < 0.001); and higher ISS (p < 0.001) were the factors that had a significant association with patient outcome (table 1-3).

***Multivariate analyses***

Presence of an underlying illness (p = 0.03), loss of consciousness for more than 24 hours (p = 0.04), spinal injury (p = 0.002), presence of multiple trauma (p = 0.01), increased ISS (p < 0.001), need for ventilator (p < 0.001), and organ failure during hospitalization (p < 0.001) were independent factors that increased the risk of poor outcome in RTI patients. In contrast, a single abdominal trauma (p = 0.006) and hospitalization in ICU were associated with improved outcome (Table 4). 

## Discussion

The present study showed that young males are most frequently affected with RTI and motorcycle is the most important cause, which is in line with previous studies. For instance, Yousefzadeh et al. showed that the number of men involved in RTI was 3.6 times the women, and about 50% of the patients were 20-44 years old. Most injuries were due to motorcycle accidents and 5.2% died in the end (10). Torabi et al. also revealed that 89.9% of the injured were male and mostly (56.8%) 16-25 years old (11). In another cross-sectional study in Tehran, most of those injured in RTI were 21-30 years old (22.3%), and mainly pedestrians (54.6%) (12). Hatamabadi et al. also expressed that majority of those who were killed in traffic accidents were male, most of which were 21-40 years old and uneducated (13). These researchers, in another study, reported 7.0% mortality rate due to RTI. 78.5% male and the majority aged 20-30 years old and most used personal cars (52.9%) (14). This higher mortality rate was due to the nature of the road they studied. Abali-Tehran is an inter-city road in Iran that has steep slopes and can be very slippery especially in rainy seasons. In addition, driving speed is much higher compared to urban streets and there are fewer motorcycles, which might justify the low rate of motorcycle accidents. In the present study, mortality due to RTI was 3.05%. Akbari et al. studied RTI in 10 provinces of Iran and concluded that mortality rate in unintentional accidents was 4% which is in line with this study. Traffic accidents with 7.51% were the most frequent cause of death (15). In addition, Yousefzadeh et al. epidemiologically evaluated effective factors in trauma patients in Rasht, Iran, and showed that 5.17% of RTI injuries result in death (10). Torabi et al. assessed motorcycle accidents and revealed 4% mortality in these patients. Their most important cause of death being head and neck trauma (11). A study by Kadivar et al. also showed that RTI was the major cause of death in unintentional accidents (16).

Despite RTI being the third most important cause of death, since it targets the younger population (mean age was about 34 years in 2001), it ranks first in the list of causes for years of potential life lost (6, 17). Controlling and decreasing RTI is not the responsibility of health care providers but informing the responsible organizations on the importance of this problem and cooperating with them to control and reduce this major cause of death can be. The statistics of this study reveal the necessity of paying more attention to emergency services and providing trauma centers and equipping them.

The reason for high mortality rate of RTI and its increase might be industrialization and broader usage of motor vehicles in recent years without improving standards for this new way of life. Reducing drug abuse, safety education, improving protective measures in working environment, rapid first aid in the location of accident, eliminating causing factors (reducing speed, putting appropriate signs on the road, etc), enforcing more restricted traffic rules, and providing rehabilitation services are among the useful measures, which can aid in prevention of accidents and therefore decrease mortality. 

The findings of logistic regression analysis showed that presence of an underlying illness, loss of consciousness for more than 24 hours, spinal injury, presence of multiple trauma, increased ISS, need for ventilator, and organ injuries during hospitalization were independent factors that increased the risk of poor outcome in RTI patients, while a single abdominal trauma and hospitalization in ICU led to improved final outcome. These results emphasize the importance of careful evaluation of these patients in ED, so that no injury goes unnoticed, because if the injuries are rapidly diagnosed and properly treated outcome can improve (18, 19). This is confirmed by the result of this study that states hospitalization in ICU leads to improved outcome. Therefore, paying attention to these patients and maintaining proper tissue perfusion during hospitalization can prevent organ disabilities and therefore poor outcome.

## Conclusion:

Based on the results of present study, underlying illnesses, loss of consciousness for more than 24 hours, spinal injury, multiple trauma, increased ISS, need for ventilator, and organ injuries during hospitalization were independent factors that increased the probability of poor outcome in RTI injuries.

## Author contribution:

All authors passed four criteria for authorship contribution based on recommendations of the International Committee of Medical Journal Editors.

## References

[1] Toroyan T (2009). Global status report on road safety.

[2] Peden M, Scurfield R, Sleet D (2004). World report on road traffic injury prevention.

[3] Organization WH (2009). World health statistics 2009.

[4] Saadat S, Soori H (2011). Epidemiology of traffic injuries and motor vehicles utilization in the Capital of Iran: A population based study. BMC public health.

[5] Ghaffar A, Hyder AA, Masud TI (2004). The burden of road traffic injuries in developing countries: the 1st national injury survey of Pakistan. Public health.

[6] Saadat S, Yousefifard M, Asady H, Jafari AM, Fayaz M, Hosseini M (2014). The Most Important Causes of Death in Iranian Population; a Retrospective Cohort Study. Emergency.

[7] Manouchehrifar M, Hatamabadi HR, Derakhshandeh N (2014). Treatment Costs of Traffic Accident Casualties in a Third-level Hospital in Iran; a Preliminary Study. Emergency.

[8] Rahimi-Movaghar V, Zarei MR, Saadat S, Rasouli MR, Nouri M (2009). Road traffic crashes in Iran from 1997 to 2007. International journal of injury control and safety promotion.

[9] Majdzadeh R, Feiz-Zadeh A, Rajabpour Z (2009). Opium consumption and the risk of traffic injuries in regular users: a case-crossover study in an emergency department. Traffic injury prevention.

[10] Yousefzadeh S, Ahmadi Dafchahi M, Mohammadi Maleksari H, Dehnadi Moghadam A, Hemati H, Shabani S (2007). Epidemiology of Injuries and their Causes among Traumatic Patients Admitted into Poursina Hospital, Rasht. Journal of Kermanshah University of Medical Sciences.

[11] Torabi A, Tarahi M, Mahmoudi GA (2009). Epidemiology of motorcycle accident in Khoramabad, Iran. Payesh.

[12] Fam M, Ghazizadeh A (2002). An epidemiological survey of lead to death road accidents in Tehran province in 1999. Scient J Kurdistan Uni Medl Sci.

[13] Hatamabadi HR, Vafaee R, Haddadi M, Abdalvand A, Soori H (2011). Necessity of an integrated road traffic injuries surveillance system: a community-based study. Traffic Inj Prev.

[14] Hatamabadi H, Vafaee R, Hadadi M, Abdalvand A, Esnaashari H, Soori H (2012). Epidemiologic study of road traffic injuries by road user type characteristics and road environment in Iran: a community-based approach.

[15] Akbari M, Naghavi M, Soori H (2006). Epidemiology of deaths from injuries in the Islamic Republic of Iran. Eastern Mediterranean Health Journal.

[16] Kadivar M, Aramesh K, Sharifi B, Asad A (2006). The prevalent causes of mortality in fars province, 2001. Med J Hormozgan Uni.

[17] Gururaj G, Uthkarsh PS, Rao GN, Jayaram AN, Panduranganath V (2014 ). Burden, pattern and outcomes of road traffic injuries in a rural district of India. International journal of injury control and safety promotion.

[18] Chini F, Farchi S, Camilloni L, Giarrizzo ML, Giorgi Rossi P Health care costs and functional outcomes of road traffic injuries in the Lazio region of Italy. International journal of injury control and safety promotion.

[19] Arhami doulatabadi A, Hedari K, Hatamabadi HR, Vafaei A (2013). Frequency of lower limb injuries and their causes among motorcycle accident admitted into Imam Hussein hospital during one year. J Saf Promot Injury Prev.

